# The Nanoparticle Stability and Microstructural Evolution of 9Cr-ODS Steel Under Fe Ion Irradiation at Elevated Temperatures

**DOI:** 10.3390/ma19020287

**Published:** 2026-01-09

**Authors:** Yaxia Wei, Wei Qian, Pengfei Zheng, Min Xu, Yifan Zhang, Jing Wang, Jiale Huang, Jintao Zhang, Bingsheng Li

**Affiliations:** 1 Center for Fusion Science, Southwest Institute of Physics, No. 5, Huangjing Road, Chengdu 610225, China; weiyaxia@swip.ac.cn (Y.W.); zhengpf@swip.ac.cn (P.Z.); 2 Institute of Modern Physics, Fudan University, Shanghai 200433, China; 3 School of Material Science and Engineering, Hefei University of Technology, Hefei 230009, China; 4 State Key Laboratory for Environment-Friendly Energy Materials, Southwest University of Science and Technology, Mianyang 621010, China; huangjiale001002@163.com (J.H.);

**Keywords:** ODS steel, nanoparticle stability, ion irradiation, transmission electron microscope

## Abstract

The stability of nanoparticles (NPs) in ODS steel is an important factor affecting their long-term service behavior. In the current work, the 9Cr-ODS steel samples were irradiated using 3.5 MeV Fe^13+^ ion irradiation up to 20 dpa at 350–650 °C, and the microstructure stability was studied using the transmission electron microscope. The correlation between the particle coarsening rate and the irradiation depth has been investigated. The results show that fine Y-Ti-O NPs undergo coarsening under irradiation at 350 and 500 °C, and the coarsening rate shows a trend of first increasing and then decreasing with the increase in depth. NP coarsening reached its peak at a certain depth, and the peak depth increased with the increase in irradiation temperature. While the coarsening was inhibited at 650 °C, almost no changes in particle size were observed, only slightly coarsening at the end of the irradiation layer. In addition, *b* = 1/2<111> type dislocation loops were dominant at 350 °C, and the formation of *b* = <100> type dislocation loops was confirmed at 500 °C. Dislocation lines were formed at 650 °C. Additionally, the segregation of Cr, O, C, Y, and Ti toward the surface in the irradiated layer was observed due to the surface effect. The stability of NPs with irradiation temperature is discussed.

## 1. Introduction

Oxide dispersion-strengthened (ODS) steel is a promising candidate for first wall and blanket structural components in future fusion reactors due to its low irradiation swelling, exceptional creep resistance, and reduced-activation characteristics [1,2,3,4]. Uniformly dispersed oxide nanoparticles (NPs) significantly enhance the mechanical properties of ODS steels at both room and high temperatures, consequently achieving a higher operational temperature limit than reduced-activation ferritic/martensitic (RAFM) steels [5,6]. Regarding radiation damage tolerance, NPs enhance defect resistance via two mechanisms: (i) NP/matrix interfaces acting as defect sinks, and (ii) grain refinement that improves grain boundary sink strength [7,8,9,10,11,12,13,14]. Although NPs generally maintain thermal stability at service temperatures, the irradiation-induced instabilities of NPs have been widely reported, mainly induced by four primary mechanisms: ballistic dissolution, Ostwald ripening, radiation-enhanced diffusion, and homogeneous nucleation. These mechanisms exhibit distinct size evolution pathways: Ostwald ripening (coarsening) versus inverse Ostwald processes (refinement, dominated by ballistic dissolution) [11,15,16,17,18].

Ballistic dissolution involves the ejection of atoms or clusters from nanoparticles via collision cascades induced by incident ions. These displaced atoms may (i) diffuse to other particles, (ii) dissolve into the matrix, or (iii) reincorporate into their original particles. Typical ballistic dissolution during neutron and ion irradiation was reported by Charry, Aydogan, and Yamashita [19]. Ballistic dissolution has a significant impact on the evolution of nanoparticles over a wide temperature range [20,21,22]. Therefore, its impact can be highlighted through low-temperature irradiation. Ostwald ripening originates from the Gibbs–Thomson effect, wherein solute solubility increases near high-curvature particle surfaces relative to low-curvature regions. This drives solute diffusion from smaller to larger particles, resulting in coarsening behavior where small particles dissolve while larger ones grow [20]. Typical Ostwald ripening during irradiation was reported by Lescoat, Ribis, and other researchers [23,24,25]. Usually, Ostwald ripening dominates during electron irradiation due to the absence of cascading. Zhang et al. [26] have reported that radiation enhanced the coarsening of NPs in 12Cr-ODS steel during electron irradiation, which shows a significant temperature and diffusion dependence.

Under specific conditions, counterbalancing effects among nanoparticle stability mechanisms can establish a dynamic metastable equilibrium. Representative studies (e.g., Kishimoto et al. on 12YWT [27], Pareige et al. on 16Cr-ODS [28], and Lescoat et al. on 18Cr-ODS steels [16]) demonstrate that Y-Ti-O nanoparticles maintain stability under 20–150 dpa ion irradiation at 300–700 °C, as verified by in situ irradiation experiments. Recently, Chen et al. revealed [29] significant irradiation stability variations in nanoparticles governed by distinct NP/matrix interface characteristics. Nanoparticles with coherent interfaces exhibit superior stability during irradiation, whereas varying interface types generate differential bias factors that directly modulate point defect absorption efficiency.

The existing studies show considerable discrepancies in nanoparticle (NP) stability, with different variations observed in size distribution, structural integrity, and chemical composition. This stems from the limited comparability across studies due to divergent irradiation conditions, specimen characteristics, and experimental parameters. Ion irradiation experiments represent the predominant methodology in such studies. Crucially, ion irradiation induces defect accumulation primarily within near-surface damage layers. Thus, surface effects must be taken into account, as they significantly modulate defect recombination dynamics and solute migration [30]. This work systematically investigates the size stability of NPs in 9Cr-ODS steel subjected to 20 dpa self-ion irradiation across temperature gradients (350–650 °C), with an emphasis on depth-resolved evolution. The study further aims to decouple the contributions of near-surface environments to the evolution of NP stability in ODS steels.

## 2. Experiment Procedures

### 2.1. Materials

The materials used in this work are 9Cr-ODS steels prepared via powder metallurgy technology, and the composition is listed in Table 1. The material is synthesized through the following steps: Firstly, pre-alloyed powder with a particle size < 40 μm was prepared using gas atomization. Secondly, pre-alloyed powder was mixed with Y_2_O_3_ powder (particle size 100–200 nm, content 0.35 wt.%) via mechanical alloying with high-purity Ar (99.99%) atmosphere and a ball-to-powder ratio of 10:1. The ball-milling speed was set as 140 r/min with a milling time of 100 h. Thirdly, the mixed powder was formed by hot forging and hot rolled to a plate with a thickness of 12 mm. Finally, the plate sample was normalized at 980–1200 °C for 1 h and then tempered at 700–800 °C for 0.5 h.

**Figure 1:**
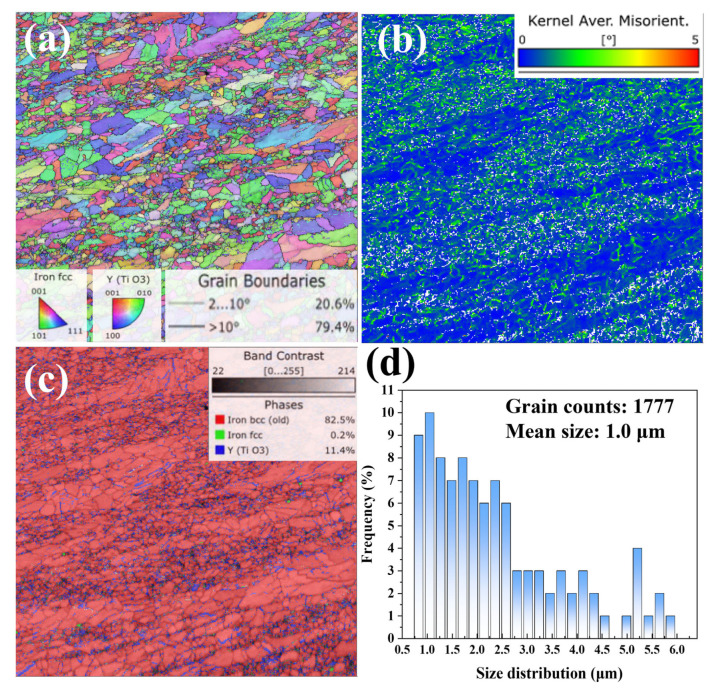
EBSD analysis of the as-fabricated 9Cr-ODS steel: (**a**) inverse pole figure, (**b**) KAM map, (**c**) phase distribution map, (**d**) grain size distribution map.

The microstructure of the as-fabricated 9Cr-ODS steel was characterized by electron backscatter diffraction (EBSD) and the results are shown in Figure 1. The inverse pole figure in Figure 1a confirms that the matrix surface is primarily composed of ferrite phase. The grain size range is from 0.5 to 6 μm and the mean size is 1.0 μm. The high values from the KAM (Kearney and Ardell-Monroe) map resulting from the EBSD analysis of 9Cr-ODS steel illustrates the significant deformation and stress of the as-fabricated ODS steels, consistent with transmission electron microscopy analysis. The phase distribution in Figure 1c reveals that a high number of nano-sized Y-Ti-O particles (about 11.4%) are distributed within 9Cr-ODS grains. However, Y-Ti-O particles are preferential to accumulate on grain boundaries.

### 2.2. Irradiation and Characterization

**Figure 2:**
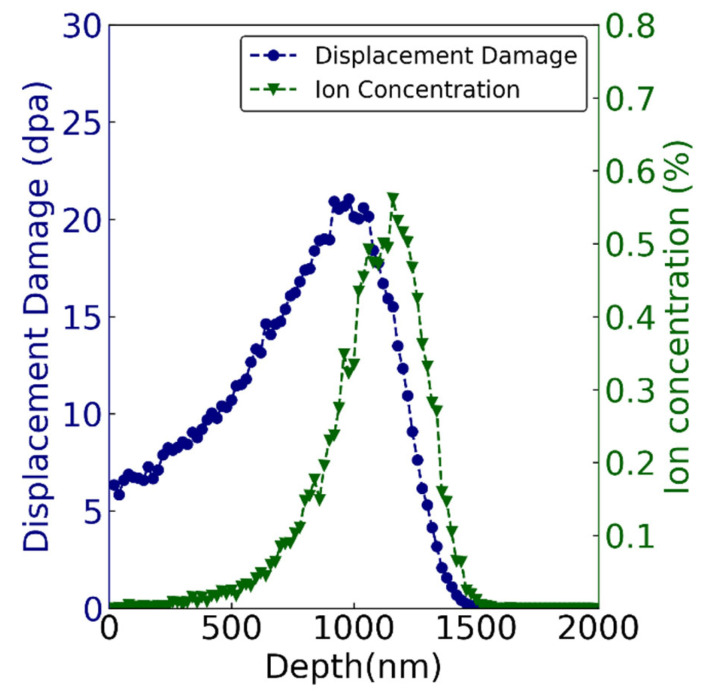
The profiles of radiation-induced displacement and ion concentration (at. %) of 3.5 MeV Fe irradiated 9Cr-ODS to a fluence of 2 × 10^16^ cm^−2^ calculated by SRIM software.

The sample for ion irradiation was 5 × 5 × 1 mm^3^ in size. Before irradiation, samples were mechanically polished to 4000 grit and then to a mirror finish using a 0.1 μm diamond spray. Polished samples were annealed at 500 °C for 2 h in vacuum (<10^−3^ Pa) to remove the residual stress during polishing. Then, 3.5 MeV Fe^13+^ ion irradiation was carried out at the 320 kV High-voltage Platform in the Institute of Modern Physics, Chinese Academy of Sciences, China. The irradiation temperatures of each group were 350 °C, 500 °C, and 650 °C, respectively. The beam flux was kept at 5 × 10^11^ ions·cm^−2^·s^−1^, and the ion fluence was 2 × 10^16^ cm^−2^. The vacuum level was better than 10^−5^ Pa in the irradiation chamber. Figure 2 exhibits the displacement profile and ion concentration calculated by SRIM-2013 software, in which the displacement threshold energy of Fe and Cr were set as 40 eV. The Kinchin-Pease estimate was used, and 10,000 ions were run. The materials’ density was set as 8.47 × 10^22^ atoms/cm^3^ (7.81 g/cm^3^), which was auto calculated by SRIM 2008 [31]. Figure 2 shows that the peak damage was ~20 displacements per atom (dpa) at about 1 μm, and the maximum depth of the damage layer was about 1.5 μm. The peak Fe ion concentration was 0.6 at.% at about 1.2 μm.

The microstructure change before and after ion irradiation was determined by transmission electron microscopy (TEM) using a Tecnai G20 (FEI, Hillsboro, OR, USA), which was operated at 200 kV with a point resolution of 0.19 nm. In order to analyze the depth distribution of the irradiation damage, cross-sectional TEM samples were fabricated by a focus ion beam (FIB) workstation (Helios-G4-CX, made by Thermo Scientific Company, Waltham, MA, USA). Initially, the voltage of 30 keV was performed and finally 5 keV was set to reduce the Ga ion-induced damage. The foils were extracted from the surface to the interior of the specimens and subsequently thinned to ~100 nm in thickness. The types of irradiation-induced dislocation loops were identified by different diffraction vectors. The element compositions of oxide particles were characterized by an Oxford energy-dispersive X-ray spectroscopy (EDS) under scanning transmission electron microscopy (STEM) condition. Bright-field (BF), dark-field (DF), and high-angle annular dark-field (HAADF) models were used to analyze oxide particles.

## 3. Results

### 3.1. Microstructure of Initial Materials

**Figure 3:**
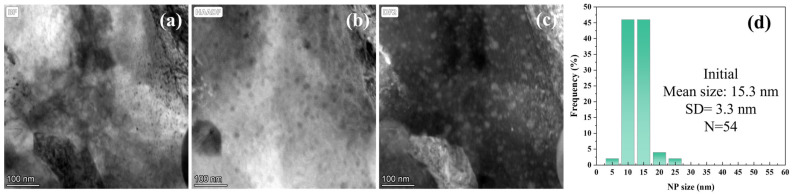
TEM image and NP size distribution of the as-fabricated 9Cr-ODS steel. (**a**) STEM-BF image, (**b**) HAADF image, (**c**) STEM-DF image, (**d**) NP size distribution.

**Figure 4:**
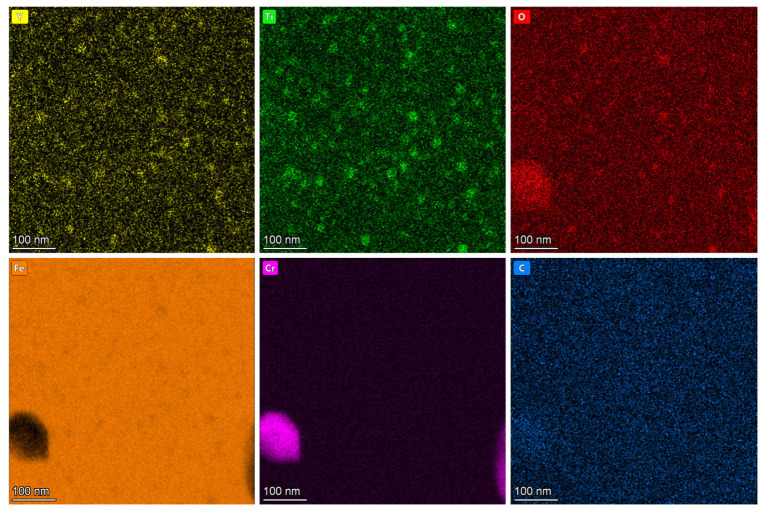
EDS element distribution results of Figure 3c.

**Figure 5:**
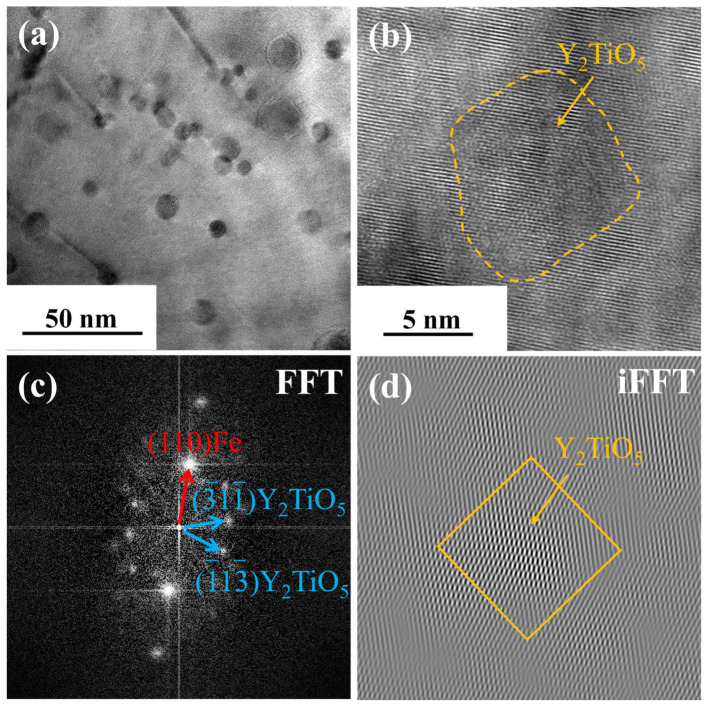
TEM image of a small NP and the corresponding FFT and iFFT images. (**a**) TEM image, (**b**) HRTEM image, (**c**) FFT image, (**d**) iFFT image.

Figure 3 characterizes the pre-irradiation microstructure of 9Cr-ODS steel, showing TEM images and NP size distribution. Figure 3a presents the STEM bright-field (BF) image. Manufacturing defects can be observed, exhibiting black spot features. However, the ring-shaped nanoparticles have poor contrast; therefore, the HAADF and DF modes were performed. Figure 3b displays the high-angle annular dark-field (HAADF) image where NPs appear dark. The STEM dark-field (DF) image is shown in Figure 3c, where NPs exhibit bright contrast. Two types of NPs were observed. In detail, most NPs are small (10–20 nm in diameter) and uniformly distributed in the Fe matrix, and minor fractions of NPs are larger, approaching 100 nm in size. Figure 3d shows the NP size distribution (excluding particles > 50 nm) statistically analyzed from the STEM-DF images. The distribution yields a mean NP diameter of 15.3 nm with a standard deviation of 3.3 nm. Figure 4 shows the EDS element distribution results of Figure 3c. It can be seen that the small NPs are composed of Y, Ti, and O. Figure 5 displays the overall morphology of the NPs and HRTEM image of a small NP in Figure 5a,b. In addition, the corresponding Fast Fourier Transform (FFT) image is shown in Figure 5c. These small NPs were identified as having Y_2_TiO_5_ structure. Figure 5d shows the inverse Fast Fourier Transform (iFFT) image of Figure 5c using the diffraction spots of ($\bar{3}1\bar{1}$) and ($\bar{1}1\bar{3}$), which highlighted the NPs, suggesting that these diffraction spots are resulting from Y_2_TiO_5_ NPs.

### 3.2. Nanoparticle Size Stability

**Figure 6:**
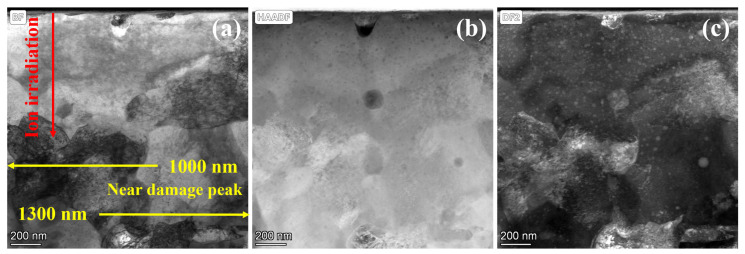
Cross-sectional TEM images showing 9Cr-ODS steel after irradiation at 350 °C. (**a**) STEM-BF image, (**b**) HAADF image, (**c**) STEM-DF image. The sample surface is on the top.

**Figure 7:**
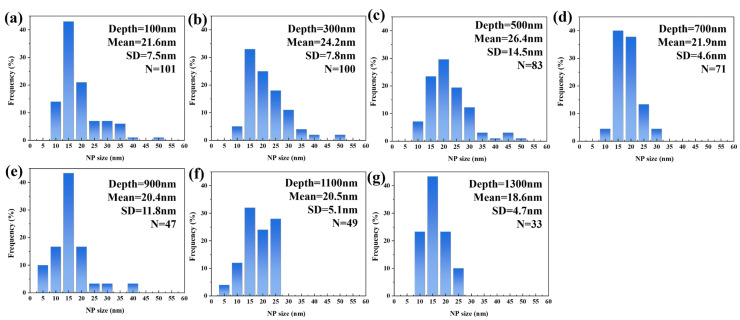
The NP size distribution with different depths from the surface after irradiation at 350 °C. (**a**) 100 nm, (**b**) 300 nm, (**c**) 500 nm, (**d**) 700 nm, (**e**) 900 nm, (**f**) 1.1 μm, (**g**) 1.3 μm.

Figure 6 displays the TEM morphology of 9Cr-ODS steel irradiated at 350 °C, with the irradiation direction indicated by the red line. Figure 6b,c reveals fine NPs within the irradiated region. Statistical analysis of precipitate size distribution was performed at 200 nm depth intervals from the surface, and the results are presented in Figure 7. Post-irradiation analysis reveals that the NPs coarsened in the damaged region. At 100 nm depth, the mean particle size was 21.6 nm, which was a 38% increase from the non-irradiated sample. At an irradiation depth of 500 nm, the particle size reaches a maximum of 26.4 nm. A subpeak was observed in the 40–50 nm range, which may be due to the statistical inclusion of a small number of larger Cr precipitates with nanoparticle-like contrast. Even when disregarding this subpeak, the average particle size remains above 20 nm at this depth. Within the 0–1 μm irradiation depth range, particles exhibit pronounced coarsening, with an average size above 20 nm. At greater depths, coarsening shows a declining trend, with the average particle size dropping to 18.6 nm at 1300 nm. Notably, particle coarsening persists even at the maximum observed depth (1300 nm). This coarsening behavior, commonly observed in irradiated ODS steels, is generally attributed to Ostwald ripening and irradiation-enhanced diffusion [17]. However, such pronounced coarsening is atypical in ODS steels; this deviation will be analyzed in subsequent sections.

**Figure 8:**
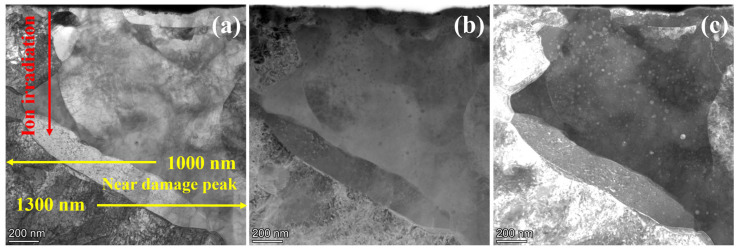
Cross-sectional TEM images showing 9Cr-ODS steel after irradiation at 650 °C. (**a**) STEM-BF image, (**b**) HAADF image, (**c**) STEM-DF image.

**Figure 9:**
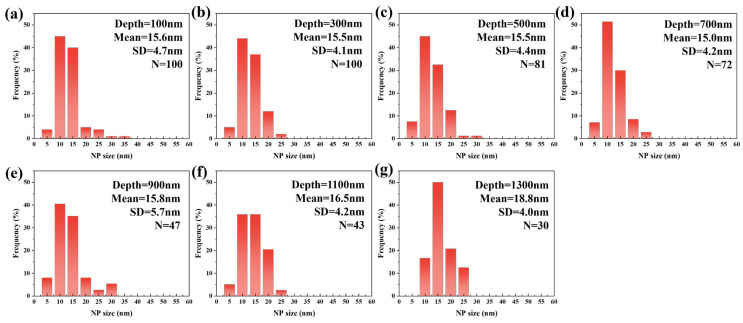
The NP size distribution with different depths from the surface after irradiation at 650 °C. (**a**) 100 nm, (**b**) 300 nm, (**c**) 500 nm, (**d**) 700 nm, (**e**) 900 nm, (**f**) 1.1 μm, (**g**) 1.3 μm.

Figure 8 presents the TEM morphology of 9Cr-ODS steel irradiated at 650 °C, while Figure 9 illustrates the corresponding statistical analysis results obtained from the STEM-DF images. The results demonstrate significant suppression of particle coarsening. At 100 nm depth, the mean particle size remained nearly unchanged at 15.6 nm, comparable to the pre-irradiation state. This size stability persisted up to a depth of 1 μm. At a depth of 1100 nm, NPs exhibited renewed coarsening, with the mean size increasing to 16.5 nm. The mean size further increased to 18.8 nm at a depth of 1300 nm, representing an approximately 20% increase compared to the non-irradiated one. Typically, nanoparticle size evolution in irradiated ODS steels results from competing mechanisms: ballistic dissolution (associated with cascade effects) and Ostwald ripening (more dominant at higher temperatures) [17]. However, the observed suppression of coarsening at 650 °C represents an anomalous behavior, suggesting the involvement of additional factors in particle size evolution.

**Figure 10:**
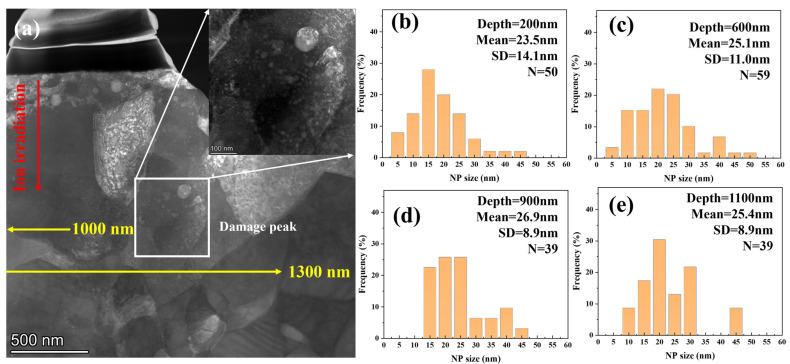
Cross-sectional TEM images showing 9Cr-ODS steel after irradiation at 500 °C and NP size distribution with different depths from the surface of 9Cr-ODS steel. (**a**) STEM-DF image, (**b**) 200 nm, (**c**) 600 nm, (**d**) 900 nm, (**e**) 1.3 μm.

**Figure 11:**
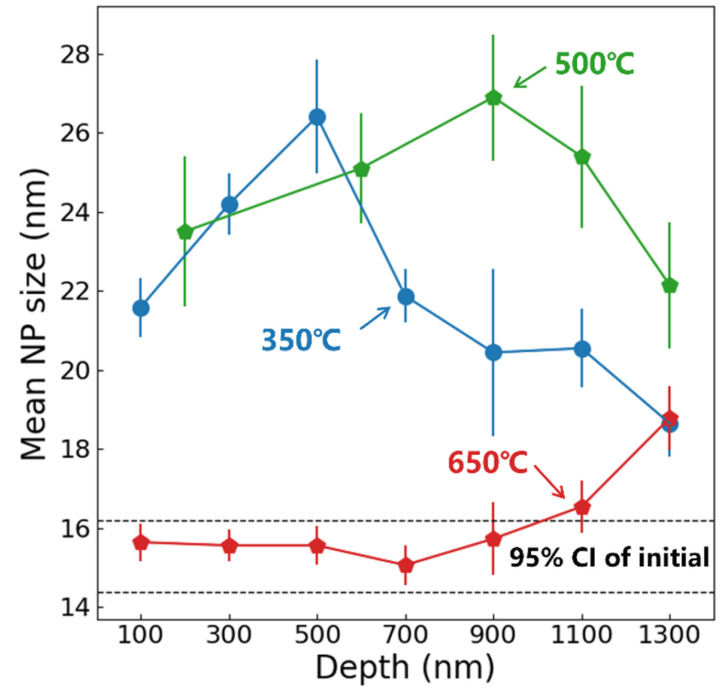
The mean NP size as a function of depth from surface in the 9Cr-ODS steel after irradiation at 350, 500, and 650 °C.

Considering that the operational temperature of ODS-RAFM steels is typically lower than 650 °C, an additional irradiation at 500 °C was conducted with the same flux and dose. However, due to the low contrast, the number of identified particles was relatively low. In order to count enough particles, the statistical range was expanded to 400 nm in shallow regions. Figure 10 shows the TEM morphology and NP size distribution at different depths. Similar particle coarsening behavior was observed compared with 350 °C; the difference is that the peak size was observed at about 900 nm, which is deeper than the sample irradiated at 350 °C. The mean NP size as a function of depth from the surface in 350, 500, and 650 °C irradiated samples is shown in Figure 11, with black dashed lines indicating the 95% confidence interval (CI) for mean NP size in unirradiated samples. The standard error (SE) was used as an error bar. At 350 °C, significant NP coarsening occurs, and the peak particle size was observed at 500 nm in depth. The coarsening of NPs was weakened at the end of the irradiation zone. At 500 °C, significant NP coarsening was also observed; the peak NP size was located at about 900 nm. In deeper areas, the coarsening of NPs was suppressed. At 650 °C, NP sizes remained comparable to unirradiated levels within the first 1 μm depth, while coarsening emerged at the extremity of the irradiation zone. This phenomenon may be related to ion irradiation surface effects and will be discussed in subsequent sections.

### 3.3. Radiation-Induced Dislocation Loops

**Figure 12:**
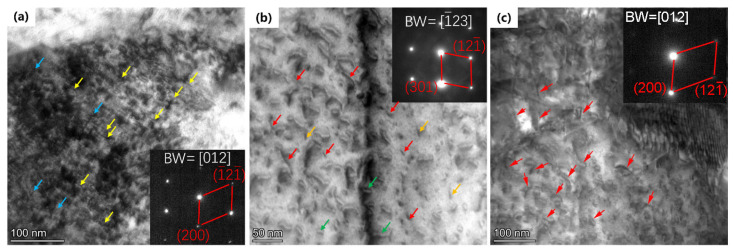
Cross-sectional TEM images showing dislocation loops in irradiated samples close to damage peak region. (**a**) 350 °C, TEM-BF; (**b**) 500 °C, TEM-BF; (**c**) 650 °C, STEM-BF image.

**Figure 13:**
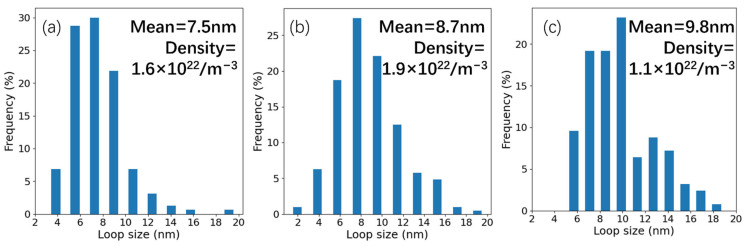
The dislocation loop size distribution in (**a**) 350 °C, (**b**) 500 °C, (**c**) 650 °C irradiated samples.

Figure 12 presents the TEM morphology of the dislocation loops in irradiated samples, and the analysis region corresponds to the yellow zone in Figure 6, Figure 8 and Figure 10. Figure 12a was recorded along the <012> direction of the Fe matrix, and Figure 12b along the <$\bar{1}23$> direction. As shown in the images, two kinds of dislocation loops were observed in the samples irradiated at 350 and 500 °C. One type exhibits an elliptical morphology, while the other appears linear. As shown in Figure 12c, a large number of dislocation lines were observed in the sample after irradiation at 650 °C. Figure 13, recorded along the <012> direction, shows the dislocation loop size distribution and number density calculated using the TEM images. The mean size indicates an increasing trend with the higher irradiation temperature. The loop mean size in the 350 °C irradiated sample was measured as 7.5 nm with a number density of 1.6 × 10^22^ m^−3^, the mean size increased to 8.7 nm, and the number density also increased to 1.9 × 10^22^ m^−3^ in the 500 °C irradiated sample. The loop size in the 650 °C irradiated sample increased to 9.8 nm on average and the number density decreased to 1.1 × 10^22^ m^−3^. This significant growth of loops is consistent with most reports [32,33,34]. It should be noted that the loops with a size smaller than 2 nm were not included in the statistics due to the resolution limitations, which may be the reason why the loop density in the 500 °C irradiated sample is higher than that in the 350 °C irradiated sample.

**Figure 14:**
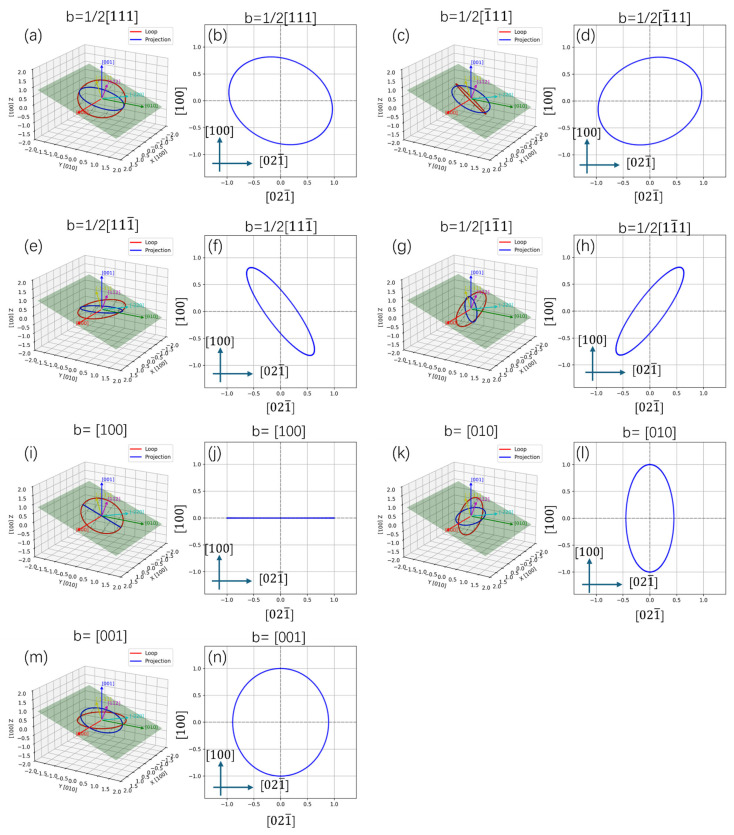
The projection relationship of dislocation loops along [012] direction of Fe matrix: (**a**,**c**,**e**,**g**,**i**,**k**,**m**) 3D structure and (**b**,**d**,**f**,**h**,**j**,**l**,**n**) 2D projection.

**Figure 15:**
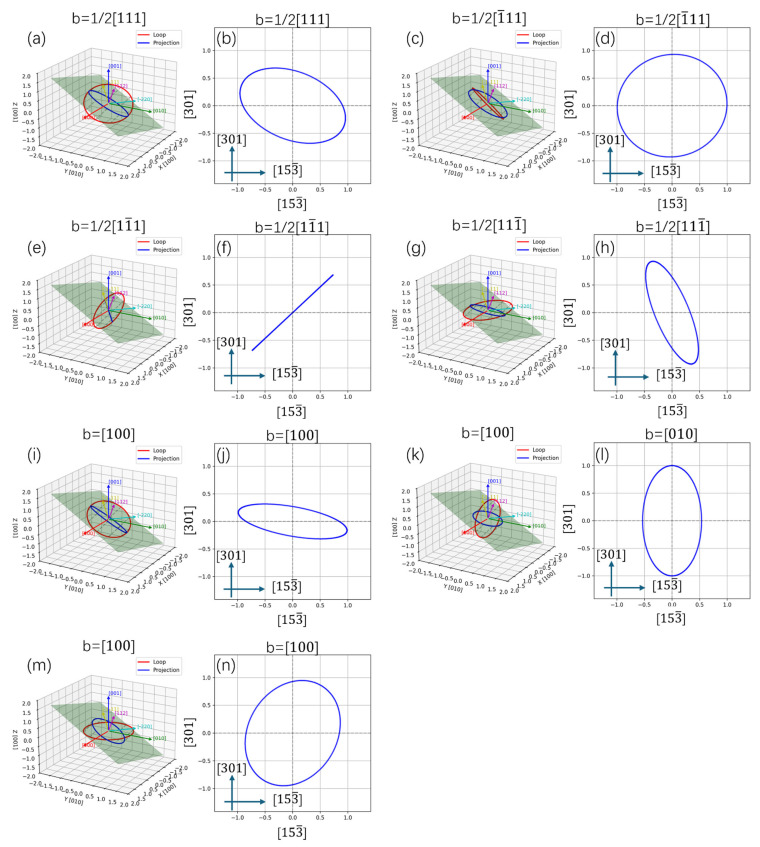
The projection relationship of dislocation loops along [$\bar{1}23$] direction of Fe matrix: (**a**,**c**,**e**,**g**,**i**,**k**,**m**) 3D structure and (**b**,**d**,**f**,**h**,**j**,**l**,**n**) 2D projection.

Usually, two kinds of dislocations can be found in the bcc-Fe: *b* = <100> type and *b* = 1/2<111> type. Since pure screw dislocations do not have a normal stress field, they do not directly interact with point defects. Assuming that all the dislocation loops are edge dislocations and exhibit a nearly circular shape, the projection relationship of dislocation loops along the [012] and [123] directions of the Fe matrix could be calculated using geometry, and the results are shown in Figure 14 and Figure 15. When observed along the <012> direction, the *b* = [100] type dislocation loop exhibits a near linear shape, and the *b* = 1/2[$1\bar{1}1$] or 1/2[$11\bar{1}$] could be near linear. Figure 14 reveals that when observed along the <123> direction, only the 1/2[$1\bar{1}1$] type dislocation loop could be linear and the *b* = [100], [010] loops could be near linear. By considering the direction of these linear dislocations, the Burgers vectors can be distinguished.

If the Burgers vector of a dislocation loop is uniformly distributed in its equivalent crystallographic direction, e.g., 1/3 of *b* = <100> type dislocation loops have a Burgers vector of *b* = [100], we can estimate the relative proportion of each type of dislocation by calculating the proportion of linear dislocations. Figure 12a shows a large number of near linear dislocations along the [$12\bar{1}$] direction of the Fe matrix (marked with yellow arrows), and similar near linear dislocations along the [$\bar{1}2\bar{1}$] direction (marked with blue arrows). These kinds of dislocation loops should be *b* = 1/2<111> type, and no *b* = <100> type linear dislocations can be confirmed. Figure 12b shows some near linear dislocations along the [$\bar{1}23$] direction of the Fe matrix, which were identified as *b* = 1/2<111> type and marked with red arrows. In addition, some linear dislocations along or perpendicular to the [301] direction were also observed, which may be *b* = [100] types, and marked with yellow and green arrows. These results suggest that *b* = 1/2<111> type dislocation loops are dominant at 350 °C, and the formation of *b* = <100> type dislocation loops is confirmed at 500 °C. Figure 12c displays the loop shape in the 650 °C irradiated sample observed along the [012] direction of the Fe matrix. It shows that almost all ellipses and a large number of dislocation lines were observed (indicated by red arrows), thereby confirming the formation of b = <100> type dislocation loops using the above method.

### 3.4. Elemental Distribution

**Figure 16:**
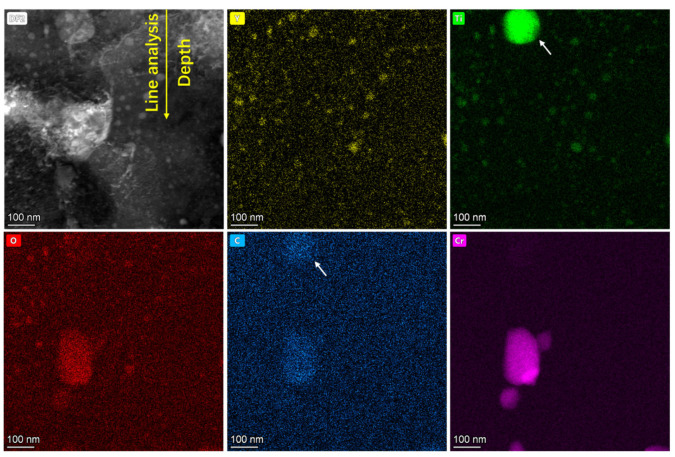
EDS element mapping near the surface in the sample after irradiation at 350 °C. TiC particles were indicated by white arrows.

**Figure 17:**
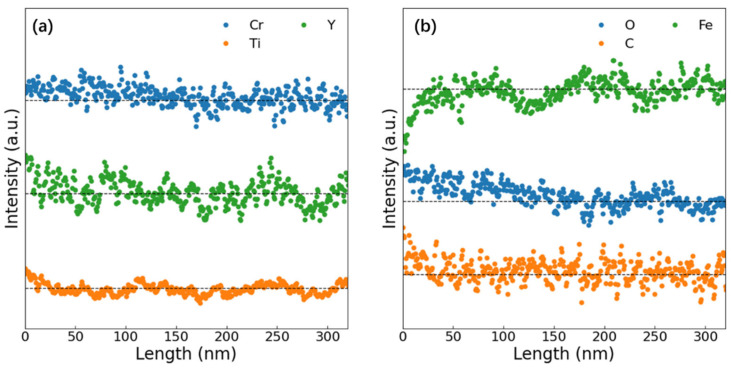
EDS element line profiles along the yellow arrow in Figure 16. (**a**) Al, Cr, Ti, Y, Zr and (**b**) Fe, O, C.

Figure 16 presents elemental mapping near the surface of the sample irradiated at 350 °C. In addition to the pre-existing Y_2_TiO_5_ nanoparticles and Cr precipitates, Ti-rich/C-rich precipitates (marked with white arrows) appear at the surface, which may be TiC phases. As EDS analysis was non-in situ, the TiC formation timing (pre-/post-irradiation) remains undetermined. Figure 17 shows EDS line profiles along the yellow arrow in Figure 16. The intensity profiles are normalized per element and do not indicate relative concentration. Dashed black lines denote average elemental concentrations in the last 100 nm. The results show that minor surface segregation of Cr, Y, and Ti and significant O enrichment and minor C enrichment at the surface were observed in Figure 17b. Correspondingly, Fe depletion occurs at the surface due to the segregation of other elements. The surface segregation observed during the irradiation may result from the influence of surface effects on the migration of point defects. Considering the high oxygen segregation near the surface, the inhibition of NP coarsening near the surface may also be related to this segregation. A detailed analysis will be presented in the next section.

## 4. Discussion

Under irradiation conditions, nanoparticle stability in ODS steels is governed by four primary mechanisms: (i) ballistic dissolution, (ii) Ostwald ripening, (iii) radiation-enhanced diffusion, and (iv) homogeneous nucleation [7,17,23]. However, these established mechanisms fail to explain the nanoparticle size evolution observed in our experiments, where NPs exhibit significant coarsening in the samples irradiated at 350 and 500 °C but inhibition of coarsening at 650 °C. This discrepancy likely arises because both Ostwald ripening and ballistic dissolution models assume material homogeneity under irradiation, without accounting for long-range solute diffusion into or out of irradiation-damaged zones. In ion irradiation experiments, damage primarily accumulates in near-surface regions, where irradiation-generated interstitial atoms and vacancies migrate under complex driving forces.

### 4.1. The Effect of the Free Surface

If the free surface can be seen as unbiased sinks, it shows no preferential absorption of defects. The interstitial atoms or vacancy flow *J* in the unit area toward the surface near a flat surface can be expressed as [35]

(1) $$J=ZDC_{0}$$

where *D* is the diffusion coefficient of interstitial atoms or vacancies, *C*_0_ is the self-interstitial atom (SIA) or vacancy concentration far from the surface, and *Z* is the sink strength of the surface for interstitial atoms or vacancies, which can be expressed as

**Figure 18:**
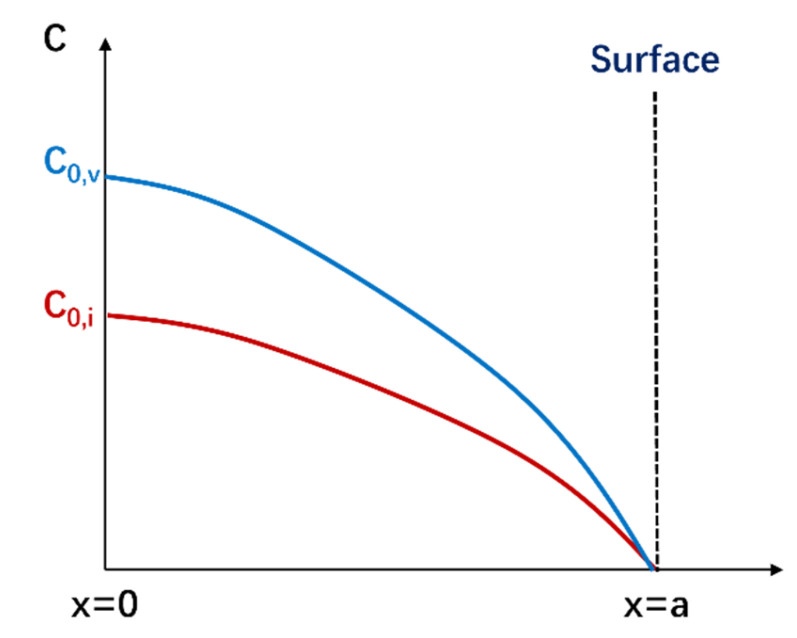
The schematic diagram of the influence of surface on SIAs and vacancy concentration.

(2) Z=kcothka

where *k* is constant and *a* is a parameter in boundary conditions, in which *C*(*x* = *a*) = 0, *C*(*x* = 0) = *C*_0_. The interstitial atoms or vacancy concentration is

(3) $$C\left(x\right)=\frac{K_{0}}{Dk^{2}}(1-\frac{\sinh kx}{\sinh ka})$$

where *K*_0_ is the defect production rate. Figure 18 shows the schematic diagram of the influence of the surface on SIAs and vacancy concentration.

**Figure 19:**
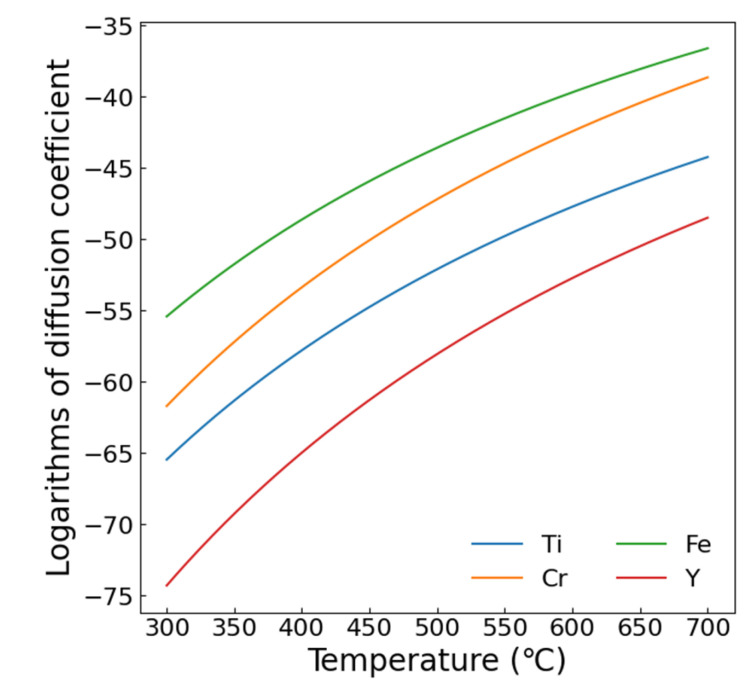
The diffusion coefficient of each element calculated using the data in Table 2.

In ODS steels, surface effects influence atomic migration through two primary mechanisms: (i) radiation-induced solute atoms (Y, Ti, etc.) migrate toward the surface as interstitial atoms and (ii) solute atoms migrate following vacancy migration due to high solute–vacancy binding energy. Table 2 lists key parameters: minimum displacement energy *T*_min_, diffusion coefficient pre-factor *D*_0_, activation energies *Q*, and binding energy of vacancy *E*_b_. Figure 19 displays element-specific diffusion coefficients derived from the data in Table 2. Cr shows the highest diffusion coefficient among the major alloying elements, explaining its pronounced segregation (Figure 16) versus minor Y and Ti segregation. This segregation reduces NP coarsening near interfaces via solute depletion. Enhanced diffusion at elevated temperatures extends the affected depth zone, shifting peak NP size to greater depths at 500 °C. At 650 °C, diffusion spans almost the entire damage zone, returning NP sizes to pre-irradiation levels. If the binding energy between solute atoms and vacancy is high enough, the strong solute–vacancy interaction will lead to the synchronous migration of vacancy and solute atoms. In this situation, the effect of vacancy migration should be considered. However, the binding energy between the Ti or Cr and the vacancies is much lower than 1 eV, and the effect of the solute–vacancy interaction should be not significant [36]. Thus, the surface segregation should be attributed to the sink strength of the free surface for SIAs.

### 4.2. Analysis of NP Size Distribution

**Figure 20:**
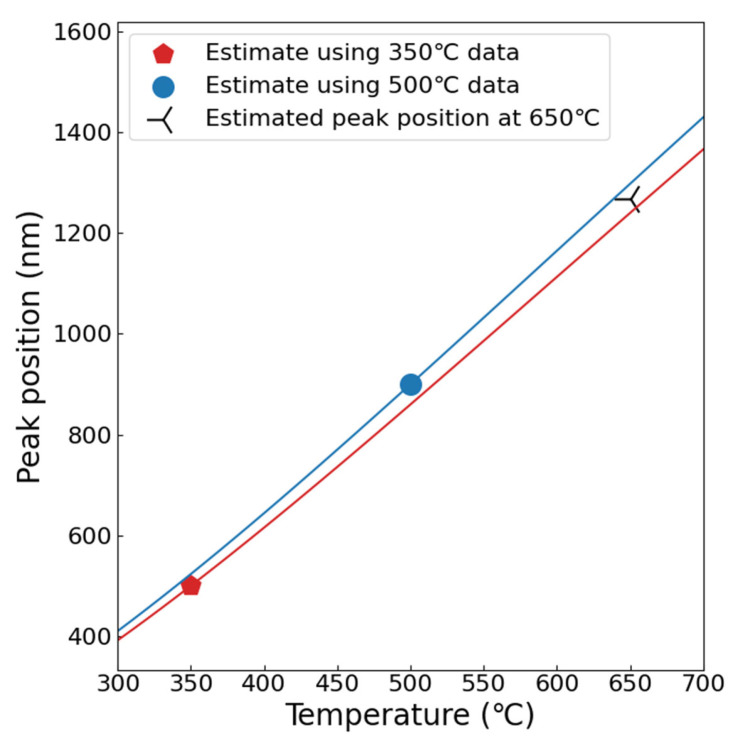
The estimated peak position as a function of irradiation temperature.

As mentioned earlier, clear peaks in particle size distribution with depth were observed in the samples irradiated at 350 and 500 °C. In the 650 °C irradiated sample, the peak position seems deeper than 1.3 μm. Suppose that the position of the size peak is controlled by the diffusion of solute atoms. That is, assuming that the depth of the diffusion influence zone is proportional to the position of the peak (*L*), based on the Gaussian solution of the diffusion equation, *L* can be expressed as

(4) $$\frac{L}{\sqrt{D^{irr}t}}=C$$

where *t* is irradiation time, $D^{irr}$ is radiation-enhanced diffusion coefficient, and *C* is constant. The radiation-enhanced diffusion coefficient $D^{irr}$ could be expressed as

(5) $$\;D^{irr}=C_{V}\exp\left(\frac{E_{V}^{f}}{k_{B}T}\right)D_{th}$$

where $E_{V}^{f}$ = 2.1 eV is the vacancy formation energy, $D_{th}$ is the thermal diffusion coefficient, *k*_B_ is Boltzmann constant, and *T* is Kelvin temperature. $D_{th}$ could be calculated using

(6) $$D_{th}=D_{0}\exp\left(-\frac{Q}{k_{B}T}\right)$$

where *D*_0_ and *Q* are given in Table 2. During the irradiation, the vacancy concentration $C_{V}$ could be estimated using

(7) $$C_{V}=\frac{R_{V}}{k^{2}D_{V}}\;$$

where $R_{V}$ is the damage rate, *k* is the sink strength, $D_{V}$ is the vacancy diffusion coefficient, and

(8) $$D_{V}=\alpha a^{2}\nu \exp\left(-\frac{E_{V}^{m}}{k_{B}T}\right)$$

where *α* = 1 is the geometric factor, *a* = 0.287 nm is the lattice parameter, and *ν* = 10^13^ s^−1^ is Debye frequency. Using these equations, the *L* as a function of *T* can be obtained as shown in Figure 20, where the red line is the estimated peak position using the data of the 350 °C irradiated sample, and the blue one is estimated using the 500 °C data. The possible peak position in the 650 °C irradiated sample was estimated to be over 1300 nm, which is consistent with our previous assessment that the diffusion influence zone almost completely covers the damaged layer.

## 5. Conclusions

This study investigated the stability of NPs in 9Cr-ODS steel under 3.5 MeV Fe ion irradiation to a dose of 20 dpa at 350, 500, and 650 °C. The key findings reveal the following:

(1) Significant nanoparticle coarsening occurred at 350 °C and 500 °C, driven by radiation-enhanced diffusion and Ostwald ripening, whereas coarsening was effectively suppressed at 650 °C, indicating a strong temperature dependence of the coarsening behavior.

(2) At 350 °C and 500 °C, nanoparticle coarsening exhibited a pronounced depth dependence, with peak coarsening observed at approximately 500 nm and 900 nm, respectively, followed by a decrease toward deeper regions. The increase in peak depth with irradiation temperature is attributed to the broadening of solute diffusion zones at elevated temperatures.

(3) Segregation of O and C was observed in the near-surface region (≤200 nm), accompanied by minor enrichment of Cr, Y, and Ti. Owing to its high diffusivity and low displacement energy, Cr dominates surface segregation, thereby reducing solute availability for nanoparticle growth and suppressing coarsening near the surface.

(4) The free surface acts as a defect sink, promoting solute depletion via radiation-enhanced diffusion, which counteracts coarsening in near-surface zones.

These results highlight the critical role of temperature-modulated diffusion and surface effects in nanoparticle stability, with implications for designing irradiation-resistant ODS steels for fusion reactors.

## Figures and Tables

**Table 1:** Chemical composition of materials used in this work (wt.%).

Fe	C	Cr	Ti	Y_2_O_3_	W
Bal.	0.13	9	0.3	0.35	1.5

**Table 2:** The minimum displacement energy *T*_min_, diffusion coefficient pre-factor *D*_0_, activation energies *Q*, and binding energy of vacancy *E*_b_ of each element.

Element	Ti	Cr	Fe	Y
*T*_min_ (eV) [37]	19	28	20	-
*D*_0_ (m^2^/s)	1.0 × 10^−6^	3.7 × 10^−3^	6.5 × 10^−5^	1.0 × 10^−5^
*Q* (eV)	2.55 [36]	2.77 [38]	2.26 [39]	3.10 [40]
*E*_b_ (eV)	0.26 [36]	0.09 [41]	-	1.45 [36]

## Data Availability

The original contributions presented in this study are included in the article. Further inquiries can be directed to the corresponding authors.
